# Comparative Genomics Analyses Support the Reclassification of Bisgaard Taxon 40 as *Mergibacter* gen. nov., With *Mergibacter septicus* sp. nov. as Type Species: Novel Insights Into the Phylogeny and Virulence Factors of a *Pasteurellacea*e Family Member Associated With Mortality Events in Seabirds

**DOI:** 10.3389/fmicb.2021.667356

**Published:** 2021-11-22

**Authors:** Eliana De Luca, Sonsiray Álvarez-Narváez, Grazieli Maboni, Rodrigo P. Baptista, Nicole M. Nemeth, Kevin D. Niedringhaus, Jason T. Ladner, Jeffrey M. Lorch, Galina Koroleva, Sean Lovett, Gustavo F. Palacios, Susan Sanchez

**Affiliations:** ^1^Athens Veterinary Diagnostic Laboratory, College of Veterinary Medicine, University of Georgia, Athens, GA, United States; ^2^Department of Infectious Diseases, College of Veterinary Medicine, University of Georgia, Athens, GA, United States; ^3^Department of Pathobiology, Ontario Veterinary College, University of Guelph, Guelph, ON, Canada; ^4^Institute of Bioinformatics, University of Georgia, Athens, GA, United States; ^5^Center for Tropical and Emerging Global Diseases, University of Georgia, Athens, GA, United States; ^6^Southeastern Cooperative Wildlife Disease Study, Departments of Pathology and Population Health, College of Veterinary Medicine, University of Georgia, Athens, GA, United States; ^7^Veterinary Medical Teaching Hospital, University of California, Davis, Davis, CA, United States; ^8^Center for Genome Sciences, United States Army Medical Research Institute of Infectious Diseases, Frederick, MD, United States; ^9^U.S. Geological Survey, National Wildlife Health Center, Madison, WI, United States

**Keywords:** bisgaard taxon 40, *Pasteurellaceae*, genomic analysis, evolution, virulence factors, phylogeny, *Mergibacter septicus*, seabirds

## Abstract

The *Pasteurellaceae* family has been associated with fatal diseases in numerous avian species. Several new taxa within this family, including Bisgaard taxon 40, have been recently described in wild birds, but their genomic characteristics and pathogenicity are not well understood. We isolated Bisgaard taxon 40 from four species of seabirds, including one sampled during a mass, multi-species mortality event in Florida, United States. Here, we present a comprehensive phenotypic and genetic characterization of Bisgaard taxon 40 and comparative genomic analysis with reference strains from the *Pasteurellaceae* family, aiming at determining its phylogenetic position, antimicrobial susceptibility profile, and identifying putative virulence factors. *In silico* multilocus sequence-based and whole-genome-based phylogenetic analysis clustered all Bisgaard taxon 40 strains together on a distinct branch separated from the other members of the *Pasteurellaceae* family, indicating that Bisgaard taxon 40 could represent a new genus. These findings were further supported by protein similarity analyses using the concatenation of 31 conserved proteins and other taxonomic approaches such as the percentage of conserved protein test. Additionally, several putative virulence factors were identified, including those associated with adhesion (capsule, *ompA*, *ompH*) and colonization (*exbD*, *fur*, *galU*, *galE*, *lpxA*, *lpxC*, and *kdsA*) of the host and a cytolethal distending toxin (*cdt*), which may have played a role in disease development leading to the mortality event. Considerably low minimum inhibitory concentrations (MICs) were found for all the drugs tested, in concordance with the absence of antimicrobial resistance genes in these genomes. The novel findings of this study highlight genomic and phenotypic characteristics of this bacterium, providing insights into genome evolution and pathogenicity. We propose a reclassification of these organisms within the *Pasteurellaceae* family, designated as *Mergibacter* gen. nov., with *Mergibacter septicus* sp. nov. as the type species. The type strain is *Mergibacter septicus* A25201^T^ (=DSM 112696).

## Introduction

The family *Pasteurellaceae* is the only member of the order *Pasteurellales* in the class *Gammaproteobacteria* (Christensen et al., 2014). Members of the family *Pasteurellaceae* have been isolated from mucosal membranes of the alimentary, respiratory, and reproductive tracts of healthy and diseased vertebrates (Christensen et al., 2003b). Although most *Pasteurellaceae* species are considered opportunistic and secondary invaders, some serve as primary pathogens and are responsible for various diseases in humans and animals (Eid et al., 2019).

Important avian pathogens are included in the *Pasteurellaceae* family with a broad clinical presentation that ranges from acute septicemia to chronic and localized infections, leading to significant morbidity and mortality in domestic and wild birds (Christensen et al., 2014). *Pasteurella multocida* subspecies *multocida* is the most common cause of fowl cholera in birds, although *P. multocida* subspecies *septica* and *P. multocida* subspecies *gallicida* may also cause fowl cholera-like disease (Christensen and Bisgaard, 2000; Christensen et al., 2014). The genus *Gallibacterium* has been reported worldwide from a broad host range among farmed and wild birds, mainly associated with reproductive disorders (Bojesen et al., 2008; Narasinakuppe Krishnegowda et al., 2020). *Avibacterium paragallinarum* is the causative agent of infectious coryza, a severe disease of the upper respiratory tract of chickens (Blackall et al., 2005; Chen et al., 2014). Other *Pasteurellaceae* species and taxa, including Bisgaard taxa, have been described in avian hosts (Christensen et al., 2003b; Blackall et al., 2005; Gregersen et al., 2009). The Bisgaard taxa represent a group of unclassified members of the *Pasteurellaceae* family isolated from several orders of birds and rodents with and without the disease (Christensen et al., 2003b, 2014). Bisgaard taxa were confirmed to belong to the *Pasteurellaceae* family by integrating comprehensive phenotypic characterization and 16S rRNA gene sequence analysis (Christensen et al., 2003b). With the advent of following next-generation sequencing (NGS) technologies, further taxonomic efforts established more definite classification for numerous Bisgaard taxa, resulting in the establishment of five new genera (*Volucribacter*, *Frederiksenia*, *Caviibacterium*, *Conservatibacter*, and *Spirabiliibacterium*) within the *Pasteurellaceae* family (Christensen et al., 2014; Korczak et al., 2014; Adhikary et al., 2018; Bisgaard and Christensen, 2019). However, the classification of some Bisgaard taxa, including Bisgaard taxon 40, is still not fully resolved, and this taxon remained unnamed (Christensen et al., 2003b).

Bisgaard taxon 40 was first isolated from the respiratory tract of a common gull (*Larus canus*) in Denmark (Christensen et al., 2003b). More recently, Bisgaard taxon 40 has been reported in rhinoceros auklets (*Cerorhinca monocerata*) with pneumonia and septicemia in Washington, United States (Knowles et al., 2019). Phylogenetic analysis based on 16S rRNA sequence revealed that Bisgaard taxon 40 was closely related to Bisgaard taxon 14 (reclassified as *Spirabiliibacter mucosae*), which was isolated from the respiratory tract of ducks, pigeons, turkeys, and pheasants with septicemia (Bisgaard and Mutters, 1986) and Bisgaard taxon 32 (reclassified as *Spirabiliibacter pneumonia*) isolated from hawks and pigeons with pneumonia and conjunctivitis (Boot and Bisgaard, 1995). These taxa (40, 14, and 32) formed a clade with *testudinistestudinis* and *Chelonobacter oris* and were classified in the Testudinis 16S rRNA group (Christensen et al., 2003b). Aside from their 16S relationships, little information is available on the genomic diversity, virulence factors, antimicrobial resistance, and evolutionary relationship between Bisgaard taxon 40 and other *Pasteurellaceae* members.

Here, we describe the first whole-genome sequence characterization for five Bisgaard taxon 40 strains, including the one isolated in a mass mortality event involving common terns (*Sterna hirundo*) and sandwich terns (*Thalasseus sandvicensis*) on the coast of Marco Island, Florida (United States) in October 2018 (Niedringhaus et al., 2021). Comparative genomic and proteomic analyses were conducted between Bisgaard taxon 40 and fifty-eight representative members belonging to thirty-three genera within the *Pasteurellaceae* family. This included the investigation of virulence genes and sequence analysis and phylogenetic relationships using core genome and targeted gene sequences. This study also aimed to investigate the presence of antimicrobial resistance genes and the susceptibility of Bisgaard taxon 40 to eighteen antimicrobial agents.

## Materials and Methods

### Bacterial Isolation, Species Identification, and Phenotypical Characterization

Brain, liver, heart, and spleen samples from three common terns were submitted to the Athens Veterinary Diagnostic Laboratory for aerobic and anaerobic bacterial culture at the University of Georgia (UGA). Tissues were rinsed with sterile phosphate-buffered saline (PBS) and processed in 3ml of brain heart infusion (BHI) enrichment broth (BD, Franklin Lakes, New Jersey, United States) using bead beater technology (Next Advance, Troy, New York, United States). After homogenization, the specimens were cultured on 5% sheep blood (BD, Franklin Lakes, New Jersey, United States), MacConkey, and Chocolate agar (Remel, San Diego, California, United States) and incubated at 42°C for 24–48h in aerobic conditions. Brucella agar (Remel, San Diego, California, United States) was also used to evaluate growth in anaerobic conditions. Matrix-assisted laser desorption ionization time-of-flight (MALDI-TOF) mass spectrometry analysis (VITEK MS - bioMérieux, Marcy-lʹÉtoile, France) was used following the manufacturer’s instructions to attempt bacterial genus and species identification. Additionally, specific bacterial traits were tested, including catalase, oxidase, urease, and indole production. As per the manufacturer’s instructions, carbon source utilization testing was performed using a GEN III MicroPlate (Biolog, Inc., Hayward, California, United States).

Four additional isolates were obtained by the U.S. Geological Survey, National Wildlife Health Center (NWHC; Madison, Wisconsin) during routine disease investigations across the United States. Information on these isolates is presented in Table 1. Strains from these four birds were isolated by touching the tissue directly onto 5% sheep blood agar plates and streaking the inoculated area of the plate for isolation. Plates were incubated at 37°C for 24–48h under aerobic conditions. Isolates originating from NWHC were identified by sequencing the V1-V3 regions of the 16S rRNA gene as described previously (Shearn-Bochsler et al., 2018). Carbon source utilization testing on NWHC isolates was performed with commercially available kits and assays, including GEN III MicroPlates (Biolog, Inc., Hayward, California, United States), Analytical Profile Index (API) 20E test strips (bioMérieux, Inc., Durham, North Carolina, United States), and 4-MU discs (Key Scientific Products, Stamford, Texas, United States).

**Table 1:** Key characters to differentiation of genera within the family.

	1	2	3	4	5	6	7	8	9	10	11	12	13	14	15	16	17	18	19	20	21
Catalase	+	+	+	+	+	+	d	d	+	d	d	−	d	d	d	d	+	+	+	−	−
Oxidase	+	+	+	+	+	+	+	d	d	+	d	+	d	+	−	d	+	−	+	+	+
β-hemolysis	+	+	+	+	+	+	d	−	−	d	+	d	−	−	−	d	+	−	−	−	−
Urease	−	−	−	−	−	−	+	−	−	−	d	−	−	−	−	−	−	−	−	−	−
Indole	−	−	−	−	−	−	d	−	+	−	−	+	−	−	−	−	−	−	+	−	d
MacConkey, growth	−	−	−	−	−	−	na	+	d	na	d	na	−	−	na	na	na	na	na	na	na
D-mannitol	−	−	−	−	−	−	−	+	−	+	+	na	−	d	d	+	+	−	−	−	+
D-sorbitol	−	−	+	−	−	+	na	d	−	na	d	na	−	d	na	na	na	d	−	−	−
L-fucose	−	−	−	−	−	−	na	d	d	−	d	na	d	d	na	−	na	na	na	na	na
D-galactose	+	+	−	−	−	+	−	d	+	−	+	na	+	+	d	+	+	na	na	+	+
D-mannose	+	+	+	−	+	+	−	d	+	−	+	na	+	+	d	+	+	+	+	na	na
Maltose	+	+	+	+	+	+	+	+	−	d	d	−	d	d	+	+	+	+	+	+	−
Sucrose	−	−	−	−	−	−	d	+	+	+	+	−	+	+	d	+	+	+	+	na	na
Trehalose	−	−	−	−	−	−	−	d	d	−	d	−	−	d	d	+	+	d	na	−	+
Dextrin	+	+	+	+	+	+	na	+	−	d	d	na	d	d	na	na	na	na	+	na	na

*Pasteurellaceae. Mergibacter: 1, A25201; 2, 27643; 3, 27576; 4, 16309; 5, 275176 (this study); 6, B301529/00/1 (Christensen et al., 2003b); 7, Haemophilus (Norskov-Lauritsen et al., 2005); 8, Actinobacillus (Christensen et al., 2003a); 9, Pasteurella (Kainz et al., 2000); 10, Mannheimia (Angen et al., 1999); 11, Gallibacterium (Christensen et al., 2003a); 12, Histophilus (Angen et al., 2003); 13, Volucribacter (Christensen et al., 2004); 14, Avibacterium (Blackall et al., 2005); 15, Aggregatibacter (Norskov-Lauritsen and Kilian, 2006); 16, Bibersteinia (Blackall et al., 2007); 17, Chelonobacter (Gregersen et al., 2009); 18, Glaesserella (Dickerman et al., 2020); 19, Frederiksenia (Korczak et al., 2014); 20, Caviibacterium (Adhikary et al., 2018); 21, Conservatibacter (Adhikary et al., 2018). Legend: d, positive or negative. na data not available*.

### Whole-Genome-Sequencing and Assembly Approach

At UGA, DNA was extracted using the DNeasy Extraction Kit (Qiagen, Hilden, Germany) following the manufacturer’s protocol. The concentration of total DNA extracted was determined using the Qubit double-stranded DNA assay kit (Thermo Fisher Scientific, Waltham, Massachusetts, United States). For the short-read sequencing using Illumina technologies (Illumina Inc., San Diego, California, United States), approximately 50ng of DNA was used for library preparation using Nextera DNA Library Prep kit (Illumina). Paired-end 300bp length reads were generated using the Illumina iSeq 100 System (Illumina Inc., San Diego, California, United States). Approximately 400ng of DNA was used for library preparation using Oxford Nanopore Technologies (ONT) library preparation SQK-RBK004 Rapid Barcoding Kit for the long-read sequencing. According to the manufacturer’s instructions, sequencing was performed on MinION with the FLOW-MIN 106 (R9.4 SpotOn) flow cell (Nanopore, Oxford, United Kingdom). Barcoded ONT sequencing reads were demultiplexed using Porechop v0.2.4^1^ and used as input into Flye v2.6 assembler (Lin et al., 2016; Kolmogorov et al., 2019) with the expected genome size parameters set at 2Mb. Illumina paired-end reads were first qualitatively checked using FastQC v0.11.8 (Wingett and Andrews, 2018) and then submitted to Trimmomatic v0.36 (Bolger et al., 2014) to remove adapters and select reads with Phred scores >30. Illumina reads were then aligned to the ONT-based resulting genome assembly using BWA v0.7.17 (Li and Durbin, 2009). Both draft ONT assembly and alignment results were then submitted to Pilon v1.23 (Walker et al., 2014) for error correction and assembly base call polishing. Assembly metrics were evaluated using the Quality Assessment Tool for Genome Assemblies v5.0.2 (QUAST; Gurevich et al., 2013). The polished assembly was then submitted to the Circlator v1.5.3 pipeline (Hunt et al., 2015) to attempt automatic assembly circularization. The final genome sequence was annotated using the NCBI Prokaryotic Genome Annotation Pipeline (PGAP; Tatusova et al., 2016; Haft et al., 2018) and deposited in GenBank under the accession number CP054053.

At NWHC, DNA was extracted from overnight cultures using a phenol: chloroform extraction method (Barbier et al., 2019). This DNA was sequenced on a Pacific Biosciences RSII at the Center for Genome Sciences, U.S. Army Medical Research Institute of Infectious Diseases (USAMRIID-CGS). Following the manufacturer’s protocol, sequencing libraries were prepared using the SMRTbell^™^ Template Prep Kit (Pacific Biosciences, Menlo Park, California, United States). DNA (~5μg) was fragmented using gTUBE (Covaris Inc., Woburn, Massachusetts, United States) to ~20kb. After DNA damage and ends repair, blunt hairpin adapters were ligated to the template, and failed ligation products were digested with ExoIII and ExoVII exonucleases. The resulting SMRTbell templates were size selected on a BluePippin system (Sage Science, Beverly, Massachusetts, United States) using 0.75% dye-free agarose cassette with 4–10kb Hi-Pass protocol and low-cut set on 4kb. Size selected templates were cleaned and concentrated with AMPure PB beads. P6 polymerase was used in combination with the C4 sequencing kit, and 360-min movies were collected. HGAP3 (Chin et al., 2013) was used for *de novo* genome assembly. Assembled genomes were visualized using Gepard v1.30 (Krumsiek et al., 2007). When evidence that a contig/chromosome was circularly complete was observed, the redundant sequence was trimmed from the ends. The breakpoint was adjusted so that the linear representation of the circular sequence began at the first base of the *dnaA* gene. Partial sequences were left unmodified. All assembled sequences were then error-corrected using Quiver v0.90 (Chin et al., 2013). Finally, all assemblies were submitted to Genbank for annotation with PGAP (Tatusova et al., 2016; Haft et al., 2018) and deposited in GenBank under the accession numbers CP022010–CP022013.

### Genome Diversity Analysis

**Figure 1:**
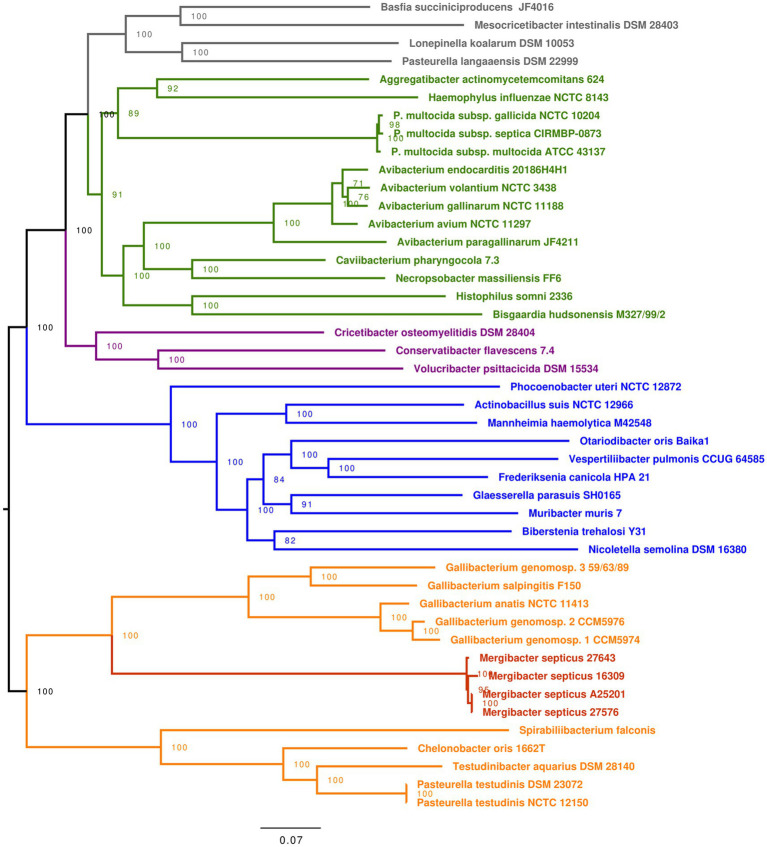
*M. septicus* whole-genome synteny based on five strains. Circular map of the comparative genome analysis performed using and the Blast Ring Image Generator software. The rings depict the percent identity between *M. septicus* genomes, represented by different colors. The second ring represents the GC content (in black).

Average nucleotide identity (ANI) and tetranucleotide signature correlation index (TETRA) scores (Richter and Rossello-Mora, 2009) were calculated using JSpecies (Richter et al., 2016) on the Progressive Mauve (Darling et al., 2004) alignment of the five *Mergibacter* whole-genome sequences obtained in this study (Table 1). Additionally, visualization of the *Mergibacter* alignment (Figure 1) was obtained using Blast Ring Image Generator (Alikhan et al., 2011).

### Phylogenomic Analyses

Three phylogenetic analyses were performed in this study based on (i) *Pasteurellaceae* bacterial core genomes (ii) 16S rRNA gene, and (iii) on a *Pasteurellaceae* housekeeping multilocus sequence (MLS). First, the complete genomes or assemblies of 58 *Pasteurellaceae* strains were downloaded from NCBI (Supplementary Table S1). The sequence alignment for the core genome-based phylogenetic analysis included 41 out of 58 *Pasteurellaceae* strains and five *Mergibacter* genomes, and it was performed using Progressive Mauve (Darling et al., 2004), followed by phylogenetic tree estimation with RAxML (Stamatakis, 2014). Then, the nucleotide sequences of the 16S rRNA and four additional housekeeping genes were manually extracted from whole genomes of these 58 *Pasteurellaceae* representatives and from *Mergibacter* strains (Supplementary Table S1). The 16S rRNA gene [1,261bp in length; from nucleotide 51 to 1,312 of *Pasteurella multocida* 20N (GenBank: CP028926)] core portion was used to construct the 16S rRNA gene tree. The *Pasteurellaceae* housekeeping multilocus sequence-based tree was constructed using the concatenated core sequences of four *Pasteurellaceae* housekeeping genes used in previous taxonomic approaches for this family (Christensen et al., 2007, 2014): *atpD* [1,152bp in length; from nucleotide 137 to 1,289 of *Pasteurella multocida* 20N (GenBank: CP028926)], *infB* [1,511bp in length; from nucleotide 714 to 2,225 of *Pasteurella multocida* 20N (GenBank: CP028926)], *recN* [1,347bp in length; from nucleotide 35 to 1,382 of *Pasteurella multocida* 20N (GenBank: CP028926)], and *rpoB* [3,918bp in length; from nucleotide 88 to 4,006 of *Pasteurella multocida* 20N (GenBank: CP028926)]. The sequence alignment for the two gene trees (16S rRNA gene, housekeeping multilocus sequence) was performed with MAFFT (Katoh and Standley, 2013) in Geneious software (version 2020.0.4), and phylogenetic trees were estimated using RAxML (Stamatakis, 2014). All trees were visualized and edited in FigTree (v1.4.4).^2^

### Taxonomic Analyses

Average amino acid identity (AAI) was calculated using MAFFT (Katoh and Standley, 2013) on the Progressive Mauve (Darling et al., 2004) alignment of the 5 *Mergibacter* sequences and 27 representatives of the *Pasteurellaceae* family, including the most genetically closed related species: *C. oris*, *Pasteurella testudines*, and *Gallibacterium* species.

The taxonomic approach known as percentage of conserved protein (POCP) was applied to the *M. septicus* type strain A25201^T^ (=DSM 112696; CP054053) and its three most closely genetically related species (*C. oris*, *P. testudines*, and *Gallibacterium genomosp. 3*) as previously described (Qin et al., 2014; Barco et al., 2020). Briefly, the predicted CDS (coding DNA sequences) for the four mentioned species were extracted using Artemis (Carver et al., 2012). BLASTp algorithm^3^ was used to find conserved proteins with an E value of less than 1e^−5^, sequence identity >40%, and comparable region of the query protein sequence >50%. Then, the POCP between two genomes was calculated as [(*C*1+ *C*2)/(*T*1+ *T*2)]×100%, where *C*1 and *C*2 represent the conserved number of proteins in the two genomes being compared, respectively, and *T*1 and *T*2 represent the total number of proteins in the two genomes being compared, respectively (Qin et al., 2014).

Finally, the taxonomic strategy for genus demarcation within the *Pasteurellaceae* family, which was previously described (Christensen and Bisgaard, 2018), was used in this study to support the reclassification of Bisgaard taxon 40 as the new genus *Mergibacter*. Briefly, the sequences of 31 conserved CDS selected (Christensen and Bisgaard, 2018) were manually extracted from *M. septicus* A25201, *C. oris*, *Pasteurella testudines*, and *Gallibacterium* species and concatenated using Geneious software (version 2020.0.4). Then, multisequence amino acid alignments were performed, and inter genus pairwise similarities were estimated with MUSCLE (Edgar, 2004).

### Identification of Virulence Factors

BLASTN v 2.2.9 (Altschul et al., 1997) was used to align the whole-genome sequences of the five *Mergibacter* isolates presented in this study against the Virulence Factors Database (VFDB; Chen et al., 2005). The parameters used for the BLAST search were ≥70% gene identity and ≥0% sequence length. Additionally, virulence factors previously described in other *Pasteurellaceae* members (Chen et al., 2014; Christensen et al., 2014; Khamesipour et al., 2014; Furian et al., 2016), including those related to capsule, outer membrane proteins (*ompA*, *ompH*, *oma87*, *plpB*, *psl*), adhesins (*ptfA*, *pfhA*, *tadD*, *hsf-1*, *hsf-1*, *hsf-2*, *ptfA*, *fimA*, *pfhA*, *tadD*) iron metabolism (*fur*, *exbD*, *tonB*, *hgbA*, *hgbB*), superoxide dismutase (*sodA*, *sodC*, *tbpA*), sialidase (*nanH*, *nanB*), toxin (*toxA*, *cdt*), and hyaluronic acid synthetase (*pm*HAS), were manually searched in all the annotated genomes using Geneious software (version 2020.0.4). The corresponding DNA sequences of virulence factors of interest were then manually extracted, and comparative analyses with those of the reference strains most closely related to *Margibacter* and/or associated with avian disease insurgence in the *Pasterellaceae* family were performed. A multisequence alignment for each virulence factor was performed with MAFFT (Katoh and Standley, 2013), and phylogenetic relatedness was estimated with RAxML (Stamatakis, 2014). Alignments and phylogenetic trees were visualized in Gingr (v 1.1.1) from the Harvest Package (Treangen et al., 2014). As a result of the comparison, heatmaps containing the percentage similarity of each virulence factor were generated using GraphPad Prism version 8.4.3 (GraphPad Software San Diego, California United States).

### Antimicrobial Susceptibility Testing and Identification of Antimicrobial Resistance Genes

Antimicrobial susceptibility testing was performed on *Mergibacter* type strain *M. septicus* A25201^T^ (DSM 112696) by broth microdilution using the Trek Sensititre system (Trek Diagnostic Systems, Cleveland, OH), followed by strip testing using Etest (bioMérieux). For the Trek Sensititre testing, individual colonies were selected from a blood agar plate and used to prepare a 0.5 McFarland suspension for inoculation of AVIAN1F plates (Thermo Fisher Scientific, Waltham, MA, United States), according to the manufacturer’s instructions. The minimum inhibitory concentration (MIC) values were visually confirmed using an inverted mirror after 24 and 48h of incubation at 42°C. The following antimicrobial compounds are included in the AVIAN1F plates: amoxicillin, ceftiofur, clindamycin, enrofloxacin, erythromycin, florfenicol, gentamicin, neomycin, novobiocin, oxytetracycline, penicillin G, spectinomycin, streptomycin, sulfadimethoxine, sulphathiazole, tetracycline, trimethoprim/sulfamethoxazole, and tylosin. Additionally, we used E-strips on Mueller-Hinton agar plates (bioMérieux) to evaluate the susceptibility for amoxicillin, amoxicillin-clavulanate, imipenem, and doxycycline, according to the manufacturer’s instructions.

We then searched for antimicrobial resistance genes (AMR) in all five whole-genome sequences analyzed, using three different databases: (i) Comprehensive Antibiotic Resistance Database (CARD) with criteria selected as perfect and strict, excluding nudge of loose hits to strict and high quality/coverage sequences^4^; (ii) ResFinder for acquired antimicrobial resistance genes, with default settings of 90% nucleotide similarity and a 60% of minimum length^5^ (Zankari et al., 2012); and (iii) AMRFinderPlus for acquired antimicrobial resistance genes, with default settings of −1.0 as the minimum identity for a blast-based hit and 0.5 as minimum coverage of the reference protein.^6^ Additionally, Plasmid Finder^7^ and COPLA plasmid taxonomic classifier^8^ databases were used to identify plasmids in all the five whole-genome sequences. Further, for the A25201 strain, three different assembly approaches were used as databases input: The Nanopore-based assembly, the Illumina reads-based assembly, and the polished assembly to investigate to which extent the assembly approach could influence the detection of plasmids.

## Results

### Growth Characteristics and Phenotype of Bisgaard Taxon 40 Strains

Growth and phenotypic analysis were performed in parallel at the University of Georgia (UGA, *n*=1) and at the National Wildlife Health Center (NWHC, *n*=4) on five bacterial isolates. In addition, a preliminary taxonomic identification of the isolates was performed by amplification and sequencing of a portion of bacterial housekeeping gene the 16S rRNA, indicating an exact match of these five isolates with sequences of the Bisgaard taxon 40 from the *Pasteurellaceae* family [i.e., AY172732.1 (Christensen et al., 2003b); MG735704.1 (Knowles et al., 2019)].

Isolate A25201 was obtained at UGA from heart and liver samples of one common tern after 48h of aerobic incubation at 42°C. Isolate A25201 grew on blood agar presenting β-hemolysis and a shiny, circular, whitish-cream colony morphology (Supplementary Figure S1). Additionally, isolate A25201 was catalase and oxidase-positive, while urease and indole tests were negative. No bacterial growth was obtained on MacConkey and Brucella agar, and Gram-negative staining rods were identified when observing A25201 isolate under the microscope (data not shown). No bacterial identification was obtained from MALDI-TOF analysis. In contrast, GEN III Microbial ID analysis revealed a mixed identification of the isolates with a low percentage of probability, including *Pasteurella canis/stomatis* (51.6%), *Histophilus somni* (15.5%), *Nicoletella semolina* (9%), and *Pasteurella pneumotropica* (9%). Acid was formed from D-glucose, D-mannose, D-maltose, D-fructose, D-galactose, D-cellobiose, methyl pyruvate, inosine, and acetoacetic acid.

Similar growth characteristics and phenotypes were observed in the four isolates (27643, 27576, 16309, and 275176) processed at NWHC (β-hemolytic, shiny, circular, whitish colonies after 48h incubation, catalase-positive, oxidase-positive, urease negative, indole negative, and no growth of on MacConkey). Surprisingly, three of the four NWHC isolates differ from isolate A25201 in their ability to utilize various carbon sources. All the phenotypic characteristics comparable between the five strains are reported in Table 2, but overall, within the characters that separate the Bisgaard taxon 40 strains from the other *Pasteurellaceae* genera are the presence of β-hemolysis and acid formation from D-mannitol and sucrose (Table 2).

**Table 2:** Strains of *Mergibacter septicus* identified in wild birds.

Strains	Host	Family	Country	Organ	Lesions	Reference
A25201^*^	Common tern (*Sterna hirundo*)	*Sternidae*	Florida (United States)	Liver/heart	Septicemia	**This study**
27643^*^	Ring-billed gull (*Larus delawarens*)	*Laridae*	Ohio (United States)	Oral swab	Not examined	**This study**
27576^*^	American herring gull (*Larus smithsonianus*)	*Laridae*	Maryland (United States)	Spleen	Septicemia	**This study**
16309^*^	Cattle egret (*Bubulcus ibis*)	*Ardeidae*	California (United States)	Liver	Not specified	**This study**
27517^*^	Common tern (*Sterna hirundo*)	*Sternidae*	Wisconsin (United States)	Lung	Septicemia	**This study**
NWHC 27363	Rhinoceros Auklets (*Cerorhinca monocerata*)	*Alcidae*	Washington (United States)	Lung	Pneumonia and Septicemia	Knowles et al., 2019
B301529/00/1	Gull, not otherwise specified	*Laridae*	Denmark (Europe)	Lung	Respiratory tract	Christensen et al., 2003b

* *Strains of Mergibacter septicus investigated by comparative genomic and virulence genes analyses (this study)*.

### Bisgaard Taxon 40 Genomic Features and Genome Diversity

For UGA strain A25201, 229 ONT reads were assembled, resulting in one single contig of 1.900Mb length with no extra leftover contigs. A total of 1.760,502 Illumina paired-end reads were aligned to the ONT resultant draft genome, and the polished assembly consisted of one single contig with a total length of 1. 887Mb. No signs of circularization and overlapping ends were identified *in silico*. For the four NWHC strains, an estimated 590-731x coverage of the genome was obtained with single-molecule real-time (PacBio) reads, and assembly resulted in a single contig. *In silico* evidence for these contigs being circularly complete was observed in strains 27517 and 27576, presenting a genome size of ~1.887Mb in length. The *de novo* assembled genomes for the other two strains, 16309 and 26643, were 1.908Mb and 1.861Mb in length, respectively. The genomic features for the five strains are summarized in Table 3.

**Table 3:** Genomic features of *Mergibacter septicus* strains.

Feature	A25201	27643	27576	16309	27517
Length (bp)	1,887,770	1,861,352	1,887,515	1,908,592	1,887,503
CDS (n)	1,693	1,612	1,679	1,670	1,678
Genes (n)	1774	1,668	1,735	1,703	1,735
Misc_Feature (n)	62	–	–	–	–
ncRNA (n)	3	1	1	1	1
Regulatory	4	–	–	–	–
Riboswitch	–	4	4	4	4
Pseudogenes	–	22	24	46	23
Repeat region (n)	1	1	1	1	1
rRNA (n)	19	19	19	19	19
tmRNA (n)	1	1	1	1	1
tRNA (n)	58	55	57	55	57
Hypothetical protein (n)	98	–	–	–	–
G+C content (%)	36.4%	41.6%	37.6%	38.5%	52.4%

*The genomes of the five Bisgaard taxon 40 strains identified and studied in this project showed high genome similarity, despite missing regions, as depicted in Figure 1. The ANI scores between the five genomes were 98.29–100%, well above the species boundary (Richter et al., 2016). Similarly, the TETRA analysis yielded correlation coefficients of 0.9985, confirming they were all isolates of the same species (Richter et al., 2016). The genomic alignment of the five isolates produced a 1,647,356bp consensus sequence with 1630; 1680; 1678; 1697; and 1677 annotated regions for strains A25201, 27643, 27576, 16309, and 27517, respectively. Of the annotated regions, 1468; 1462; 1460; 1479 and 1459 were, respectively, confirmed as protein-coding sequences (CDS)*.

### Bisgaard Taxon 40, the New *Pasteurellaceae* Genus *Mergibacter*

**Figure 2:**
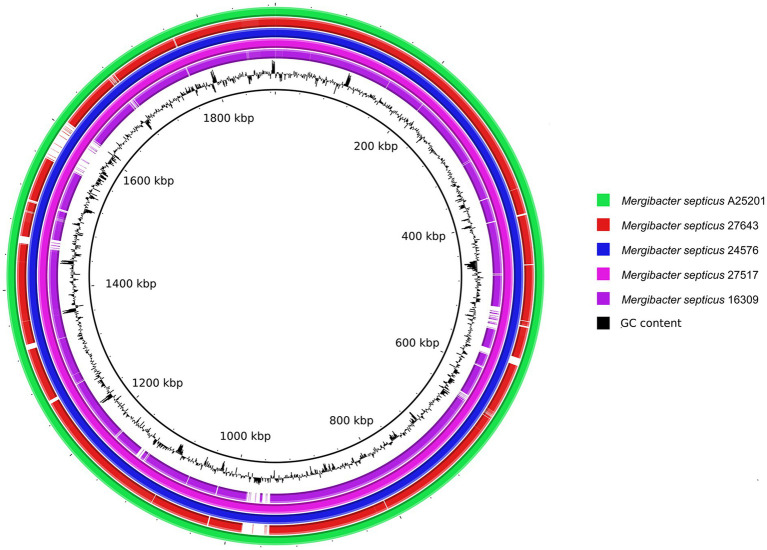
Core genome-based phylogeny of *Mergibacter* within the *Pasteurellaceae* family. The sequence alignment for the core genome-based phylogenetic analysis was obtained using Progressive Mauve (Darling et al., 2004), and phylogenetic tree estimation was done with RAxML (Stamatakis, 2014). The tree was visualized and edited in FigTree (v1.4.4; http://tree.bio.ed.ac.uk/software/figtree). Scale bar represents average number of nucleotide substitutions per site.

The genetic relatedness of the five strains of this study with 41 representative members of the *Pasteurellaceae* family was assessed by whole-genome sequence alignments. The highest inter-genus nucleotide similarity percentages were 69.2–72.9% with *Pasteurella*, 70% with *Frederiksenia*, 70–70.4% with *Mannheimia*, 70.4–70.5% with *Bibersteinia*, 70.6–70.7% with *Actinobacillus*, 70.6–72.4% with *Avibacterium*, 70.9–71.1% with *Caviibacterium*, 70.9–71.1% with *Chelonobacter*, 71% with *Glaesserella*, 72.4–72.6% with *Haemophilus*, 72.7–73.0% with *Histophilus*, 72.8–72.9% with *Conservatibacter*, 73.3–73.4% with *Volucribacter*, and 73.6–75.4% with members belonging to *Gallibacterium* genus. A core genome-based phylogenetic analysis divided *Pasteurellaceae* into five main clades and consistently placed the five Bisgaard taxon 40 strains as a distinct, well-defined terminal monophyletic group (Figure 2). Although closely related, Bisgaard taxon 40 and genera *Gallibacterium*, *Pasteurella*, and *Chelonobacter* form differentiated clades under a common node indicating the identity of Bisgaard taxon 40 as a new genus.

Three different taxonomic tests were performed to determine the integrity of this finding. First, an AAI analysis using 764 CDS conserved across *Pasteurellaceae* showed that the five Bisgaard taxon 40 isolates shared the highest amino acid similarity percentages with *Gallibacterium*, ranging from 67.78–68% with *G. genomosp. 2* to 69.8–70% with *G. genomosp. 3*. The AAI scores observed here are far below the AAI thresholds for separating potential genera in other bacterial families (Konstantinidis and Tiedje, 2007; Sangal et al., 2016), further supporting the belonging of the five subject strains to a new genus. Second, a POCP taxonomic analysis was performed on Bisgaard taxon 40 and its three most closely genetically related species (according to core genome phylogeny). Surprisingly, the results obtained by testing the taxonomy of the Bisgaard taxon 40 strains with this popular approach were inconclusive. The current POCP value proposed as a genus boundary for most prokaryotic groups is <50% (Qin et al., 2014). However, all the POCP values obtained in this study were above the 50% threshold even between members of supposedly genetically distant genera, such as *Chelonobacter* and *Pasteurella*, that shared the highest percentage (~79%) of conserved proteins in this study. Indeed, POCP values of 68.34, 63.28, and 60.81% were obtained between Bisgaard taxon 40 strain A25201 and *G. genomosp. 3*, *C. oris*, and *P. testudinis*, respectively (Supplementary Table S3). In addition, strain A25201 had a lower shared POCPs with the representative strains of its closest genera (*Gallibacterium*, *Chelonobacter*, and *Pasteurella*) than those genera members between each other. Finally, we performed a *Pasteurellaceae*-specific taxonomic approach based on the AAI of 31 randomly selected conserved protein sequences (Christensen and Bisgaard, 2018). Our results showed that the similarity percentages calculated between Bisgaard taxon 40 A25201 and the closest *Pasteurellaceae* members ranged from 68.2% with *P. testudinis* to 69.59% with *G. genomosp*. 3, well below the highest inter genus pairwise similarity (88%) identified between *Pasteurellaceae* members (Christensen and Bisgaard, 2018; Supplementary Table S4). These results recognize once again Bisgaard taxon 40 as a new genus within the *Pasteurellaceae* family.

Hence, based on this taxonomic evidence and the core genome phylogeny described above, we renamed Bisgaard taxon 40 as genus *Mergibacter*, with *M. septicus* type strain A25201 (DSM 112696; CP054053) as a type strain.

### 16SrRNA-Based Phylogeny Is Not Accurate for Genera Classification in *Pasteurellaceae*

**Figure 3:**
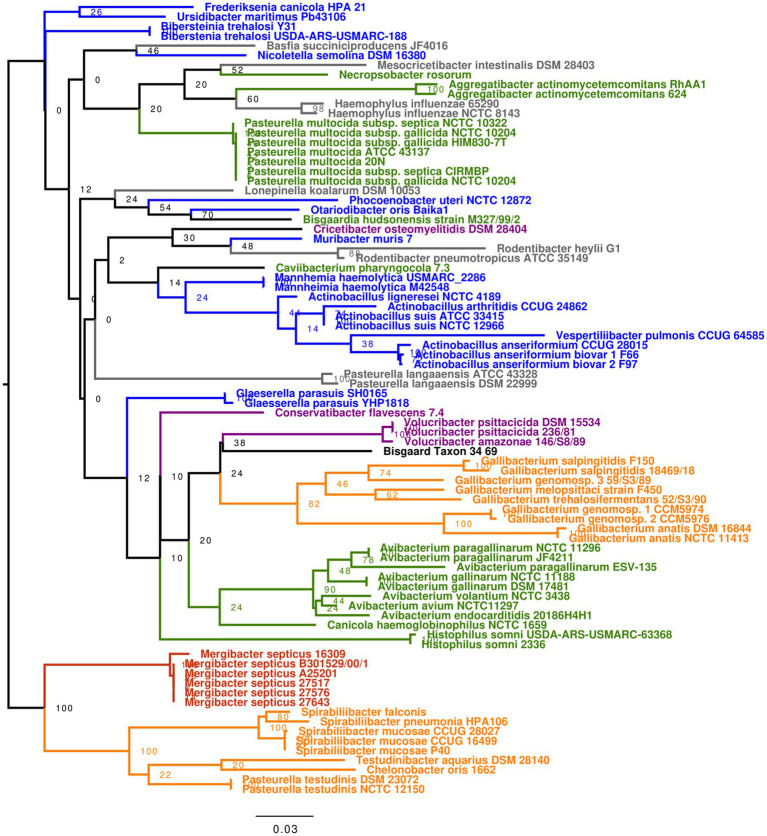
16S rRNA gene-based phylogeny of 82 *Pasteurellaceae* members including five *M. septicus* strains. The sequence alignment for the 16S rRNA-based phylogenetic analysis was performed using MAFFT (Katoh and Standley, 2013) in Geneious software (version 2020.0.4), and phylogenetic trees were estimated using RAxML (Stamatakis, 2014). All trees were visualized and edited in FigTree (v1.4.4; http://tree.bio.ed.ac.uk/software/figtree).

We tested the utility of 16S-based taxonomy to differentiate *Mergibacter* from other *Pasteurellaceae* genera. New sequence similarity and phylogenetic analyses were performed by using the 16S sequences extracted from the 58 *Pasteurellaceae* species and the five *Mergibacter* strains (Supplementary Table S1). In addition, 19 16S rRNA sequences downloaded from NCBI were included, to represent also some *Pasteurellaceae* species for which a whole-genome sequence was not publicly available (Supplementary Table S2). The five *M. septicus* strains showed high sequence similarities among each other, with nucleotide identities of 99.5–100% for the 16S rRNA gene. The highest nucleotide identities between *Mergibacter* and other *Pasteurellaceae* species were found with *P. testudinis* (92.2%), *Spirabiliibacter pneumoniae* (91.3–91.4%), *Spirabiliibacter mucosae* (91.8–92%), and members belonging to the genus *Gallibacterium* (91–92.3%). In our 16S rRNA-based phylogenetic tree, the five *Mergibacter* strains appeared on a distinct terminal group separated from its closest relatives by good bootstrap support values (Figure 3). This finding further supports *Mergibacter* as a new genus and the capacity of 16S-based taxonomy to identify *Mergibacter* representatives among other *Pasteurellaceae*. Still, some differences were observed between the core genome- and the 16S-based phylogeny estimated in this study. First, we observed that the support values for most of the nodes of the 16S were very low (in many cases <50%), even when 19 additional sequences were included in the study. In fact, the only terminal nodes that showed bootstrap values of 100% were found in the clade that separates *Mergibacter* from the other *Pasteurellaceae* genera (Figure 3). Second, the core genome-based phylogeny classified the *Pasteurellaceae* into five main clades. This topology is not as clear in the 16S tree. Additionally, the core genome phylogenetic analysis placed *Gallibacterium* as *Mergibacter* closest genus (Figure 2), while 16S-based phylogeny placed *Gallibacterium* in a very distant clade (Figure 3). This indicates that although the amplification and subsequent sequencing of the 16S rRNA gene are adequate to identify *Mergibacter*, this gene may not be the best representative to estimate phylogenetic relatedness among the members of the *Pasteurellaceae* family.

**Figure 4:**
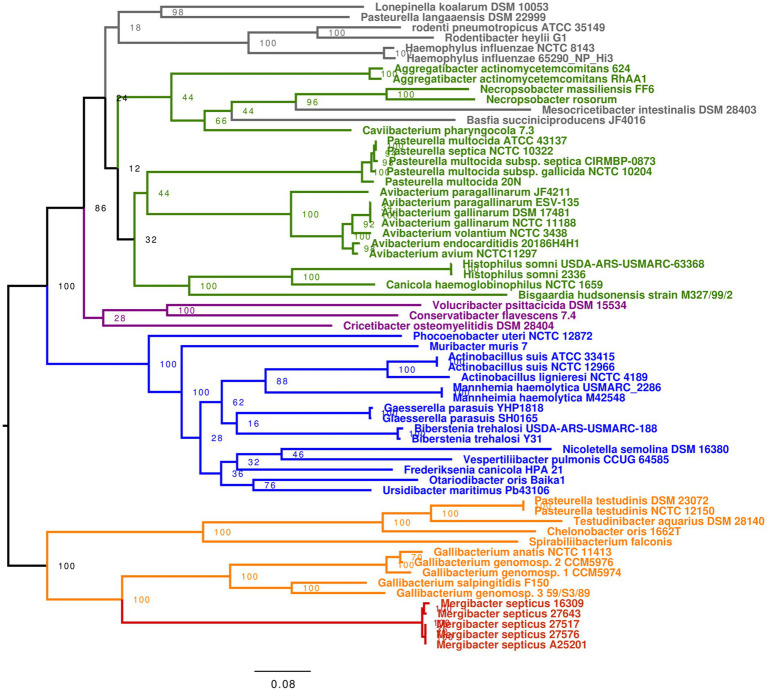
MLS-based phylogeny of 63 *Pasteurellaceae* members including five *M. septicus* strains. For each strain, core sequences of *atdP*, *infB*, *rpoB*, and *recN* genes were selected and concatenated to produce a multilocus sequence or MLS. The MLSs alignment was performed using MAFFT (Katoh and Standley, 2013) in Geneious software (version 2020.0.4), and phylogenetic trees were estimated using RAxML (Stamatakis, 2014). All trees were visualized and edited in FigTree (v1.4.4; http://tree.bio.ed.ac.uk/software/figtree).

Considering the findings mentioned above, we decided to investigate a cost-effective alternative method for accurately identifying *Mergibacter* and classifying *Pasteurellaceae*. Housekeeping genes *infB*, *recN, atpD*, and *rpoB* have been used for this purpose in a previous study (Christensen et al., 2004). We extracted these four genes from the same 63 genomes (Supplementary Table S1) used in the 16S rRNA-based phylogeny, and for each of the genomes, we concatenated the cores of the four genes to create a representative multilocus sequence (MLS). Once again, the five *M. septicus* showed high similarities among each other, with nucleotide identities of 99.2–99.9% for the MLS, and the closest relatedness was found with members of the *Gallibacterium* genus, with nucleotide identity values of 80.2–83.3%, 76.6–82.7%, 62.8–64.8%, 82.2–83.5%, and 79.5–80.7%, respectively. The MLS-based phylogenetic tree showed a similar topology to the core genome-based tree with five distinct clades, and *Mergibacer* as a terminal monophyletic group in a cluster containing closest genera *Gallibacterium, Chelonobacer, Spirilibacterium, and Testudinibacter* (Figure 4). Although higher values are appreciated in the MLS tree, these are still below those observed in the core genome tree and genera *Mesocribacter* and *Basfia* appear misplaced (Figure 4). This suggests that our MLS approach is better than conventional 16S rRNA-based taxonomic methods but still inferior to core genome-based phylogeny.

### Comparative Analysis of *Mergibacter* Virulence Genes

**Figure 5:**
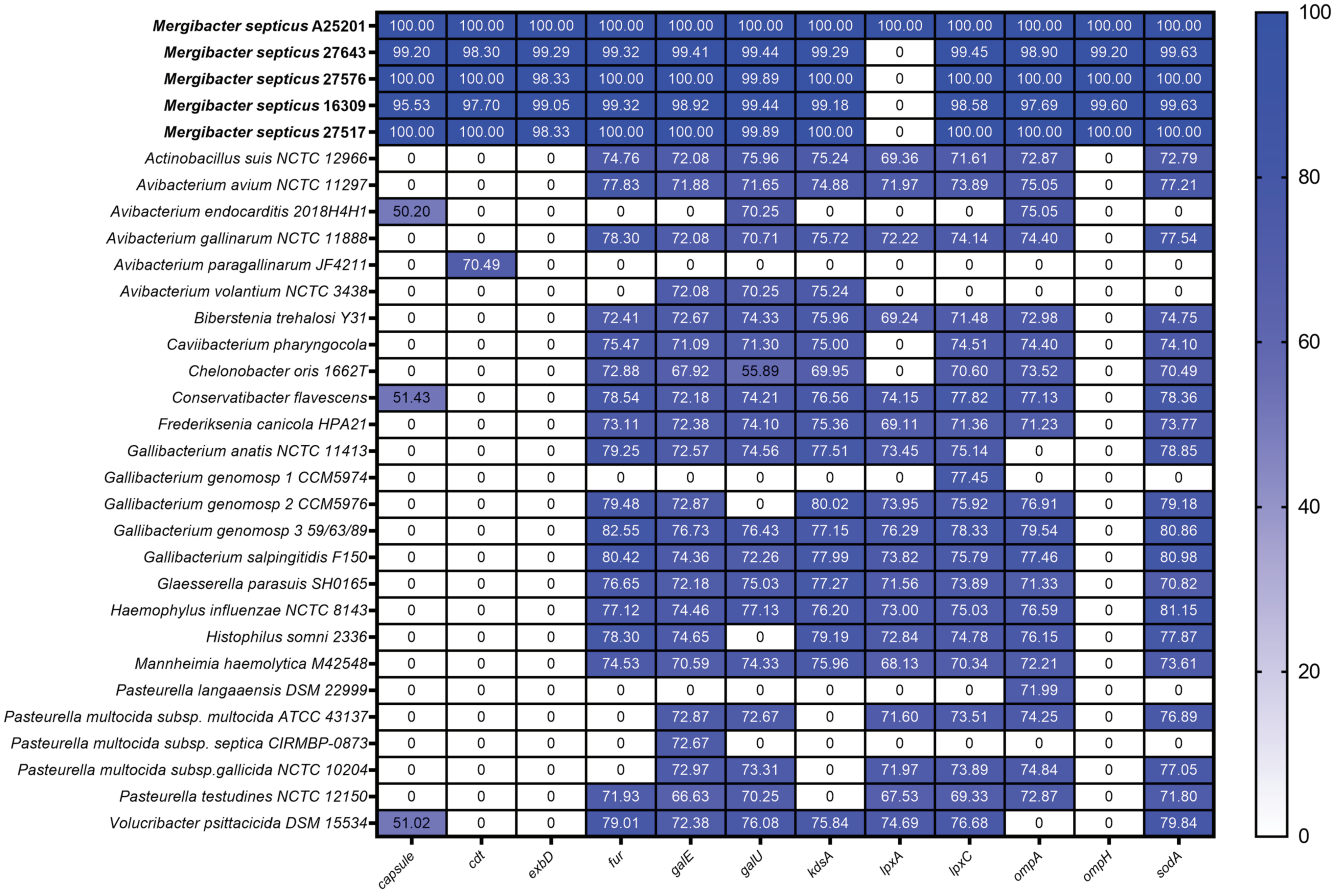
Presence and absence of virulence factors in *Mergibacter septicus* and other *Pasteurellaceae* avian pathogens. The heatmap was generated based on the similarity of each virulence gene using GraphPad Prism version 8.4.3. The color key on the right indicates percent gene similarity. Cells in white correspond to absent genes.

**Figure 6:**
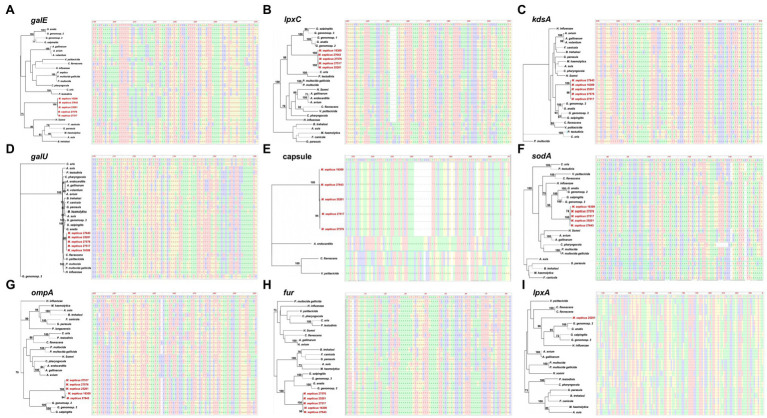
Phylogenetic relationship of virulence factors; galE **(A)**, lpxC **(B)**, kdsA **(C)**, galU **(D)**, capsule **(E)**, sodA **(F)**, ompA **(G)**, fur **(H)** and lpxA **(I)**; between *Mergibacter septicus* (in red) and other Pasteurellaceae avian pathogens (in black). A multisequence alignment for each virulence factor was performed with MAFFT (Katoh and Standley, 2013), and phylogenetic relatedness was estimated with RAxML (Stamatakis, 2014). Alignments and phylogenetic trees were visualized in Gingr (v 1.1.1) from the Harvest Package (Treangen et al., 2014).

Twelve putative virulence factors were identified in the genome of *M. septicus* type strain A25201 (Table 4), including genes encoding: a putative capsule (capsule), outer membrane proteins (*ompA*, *ompH*), a cytolethal distending toxin (*cdt*), iron metabolic proteins (*exbD*, *fur*), a superoxide dismutases (*sodA*), and proteins involved in lipooligosaccharide (LOS) synthesis (*galU*, *galE*, *lpxA*, *lpxC*, and *kdsA*). Because virulence factors are essential for establishing an infected host and provide insight into the evolutionary characteristic of bacteria (Parkhill et al., 2003), we performed a comparative analysis of each virulence factor identified in *M. septicus* A25201 with those of the other *Mergibacer* and other *Pasteurellaceae* avian pathogens. *M. septicus* A25201 presented 1 virulence factor (LOS synthesis *lpxA* gene) missing in the other isolates of this new genus but present in other *Pasteurellaceae* (Figures 5, 6). Nine virulence genes (*galU*, *galE*, *lpxC*, *lpxA*, *kdsA*, *ompA*, *sodA*, *kdsA*, and capsule) were shared by all *Mergibacter* members and most of the other *Pasteurellaceae* genera, and two putative virulence genes rarely found in common *Pasteurellaceae* avian pathogens, *cdt* and *ompH*, were found in all *Mergibacer*. For most of the shared virulence genes, *Mergibacter* showed the highest sequence similarity with members of the genus *Gallibacterium* (Figure 5), and in all resultant phylogenetic trees, it clustered on a different branch, which descended from the same node of *Gallibacterium* members (Figure 6).

**Table 4:** Virulence factors identified in *Mergibacter septicus strains*.

Process or enzyme	Gene	Protein
Capsule biosynthesis	capsule	capsule biosynthesis protein
Outer membrane proteins	*ompA*	porin OmpA
	*ompH*	OmpH family outer membrane protein
Toxin	*cdt*	cytolethal distending toxin protein
Iron metabolism	*exbD*	biopolymer transporter ExbD
	*fur*	ferric iron uptake transcriptional regulator
Superoxide dismutases	*sodA*	superoxide dismutase family protein
LOS (lipooligosaccharide) synthesis	*galU*	UTP--glucose-1-phosphate uridylyltransferase GalU
	*galE*	UDP-glucose 4-epimerase GalE
	*lpxA*	acyl-ACP--UDP-N-acetylglucosamine O-acyltransferase
	*lpxC*	UDP-3-O-acyl-N-acetylglucosamine deacetylase
	*kdsA*	3-deoxy-8-phosphooctulonate synthase

### *Mergibacter* Antimicrobial Resistance Phenotype and Potential ARGs and ARGs-carrying Plasmids

The antimicrobial resistance phenotype of *M. septicus* type strain A25201 (DSM 112696; CP054053) was tested in this study. Isolate A25201 presented considerably low MICs for all the drugs tested, including penicillin, amoxicillin, and tetracycline (Table 5). Furthermore, no antimicrobial resistance genes were found in all the five *Mergibacter* genomes after searching on the ResFinder and ARMFinder databases. Of note, CARD database analysis identified sequence variants (92.6% of nucleotide identity) described in *Escherichia coli* elongation factor Tu (EF-Tu), conferring resistance to pulvomycin (Zeef et al., 1994). Comparison with phenotype for pulvomycin was not possible as it is not included in commercially available antimicrobial susceptibility panels. No plasmids were identified using Plasmid Finder and COPLA plasmid taxonomic classifier databases.

**Table 5:** Antimicrobial susceptibility profile of *Mergibacter septicus* strain A25201 against 21 antimicrobials agents using Trek Sensititre and E-strips.

Antimicrobic	MIC (μg/ml; Trek Sensititre) Liver	MIC (μg/ml; Trek Sensititre) Heart	MIC (μg/ml; E-strips) Liver	MIC (μg/ml; E-strips) Heart
Amoxicillin	≤0.25	≤0.25	≤0.38	≤0.38
Amoxicillin-clavulanate			≤0.38	≤0.25
Ceftiofur	≤0.25	≤0.25		
Clindamycin	≤0.5	≤0.5		
Enrofloxacin	≤0.125	≤0.125		
Doxycycline				0.25
Imipenem				0.75
Erythromycin	0.5	0.5		
Florfenicol	≤1	≤1		
Gentamicin	≤0.5	≤0.5		
Neomycin	≤2	≤2		
Novobiocin	≤0.5	≤0.5		
Oxytetracycline	≤0.25	≤0.25		
Penicillin G	0.25	0.25		
Spectinomycin	≤8	≤8		
Streptomycin	≤8	≤8		
Sulfadimethoxine	≤32	≤32		
Sulphathiazole	≤32	≤32		
Tetracycline	≤0.25	≤0.25		
Trimethoprim/Sulfamethoxazole	≤0.5	≤0.5		
Tylosin	≤2.5	≤2.5		

*Legend: MIC, minimum inhibitory concentration; n.a., interpretation not available*.

## Discussion

Bisgaard taxon 40, initially isolated from respiratory tract lesions of a gull and classified in the *Pasteurellaceae* family, represents a recently discovered bacterium causing disease in wild birds (Christensen et al., 2003b). In recent years, increasing evidence indicates that Bisgaard taxon 40 is associated with a wide range of pathological changes leading to mass mortality events in wild birds (Knowles et al., 2019). The present study presents a comprehensive phenotypic and genetic characterization of 5 isolates of Bisgaard taxon 40 and a comparative genomic analysis between them and 58 reference members belonging to 31 genera within the *Pasteurellaceae* family. Our data demonstrated that Bisgaard taxon 40 represents a new genus that we nominated as *Mergibacter*, with the type species *M. septicus* and type strain *M. septicus* A25201 (DSM 112696; CP054053). Additionally, this project identified twelve potential virulence factors putatively involved in *Mergibacter* pathogenesis.

A new genus of bacteria is primarily recognized by a distinct phenotype that separates it from its neighbors (Christensen et al., 2014). Phenotypical and biochemical characteristics, including catalase, urease, indole, ornithine decarboxylase, phosphatase tests, growth on MacConkey, and carbon source utilization tests, are frequently the most frequently used for genera differentiation within the *Pasteurellaceae* family (Blackall et al., 2005; Christensen et al., 2007). The five *Mergibacter* strains studied here shared similar phenotypic characteristics, such as β-hemolysis and a shiny, circular, whitish-cream colony morphology on blood agar. Within the features which separate *Mergibacter* strains from other *Pasteurellaceae* genera were the presence of β-hemolysis and acid formation from D-mannitol and sucrose (Christensen et al., 2003a). However, this limited number of phenotypic differences and the high phenotypic divergence presented by the different species of the family made *Mergibacter* phenotype-based classification very difficult. Furthermore, misidentification based upon phenotypic characterization represents a common problem among taxa of the *Pasteurellaceae* (Christensen et al., 2007).

The arrival of new genetic-based classification methods for the *Pasteurellaceae* family (Christensen et al., 2003a) has resulted in the discovery of additional genera and the reclassification of some of the original members in recent years, including *Pasteurella*, *Haemophilus*, and *Actinobacillus*. The availability of these genomes provided the opportunity to complete comprehensive phylogenetic and comparative analyses, which identified molecular signatures, commonly shared by closely related species within the family *Pasteurellaceae* (Naushad et al., 2015). Here, in addition to the phenotypic characterization, core genome and targeted gene analyses were used to investigate the genetic variation between different *Mergibacter* isolates and also its classification within the *Pasteurellaceae* family. High sequence similarities were observed across the five *Mergibacter* isolates in this study collected from four different bird species, indicating that although genetically similar, *Mergibacter* can infect a broad range of wild bird species within the Aequorlitornithes clade. Additionally, the five *Mergibacter* strains consistently clustered together forming a well-separated terminal monophyletic group in all phylogenetic trees built in this study (16S, MLS, core genome, and virulence genes) despite not known common geographic or environmental associations (e.g., eastern and western United States vs. Scotland), reflects the potential spread of this bacteria *via* bird migration patterns as observed for known viral and bacterial pathogens such as West Nile Disease virus and Salmonella (Jourdain et al., 2007).

Classic molecular taxonomic approaches corroborated the phylogenetic evidence that Bisgaard taxon 40 is a new genus within the *Pasteurellaceae* family. One of the proposed molecular methods to differentiate bacterial genera is based on the average amino acid identity (AAI) of the conserved genes between subject species (Konstantinidis and Tiedje, 2007), with a range of AAI values (65 to 72%) as the threshold to define a different genus. *Mergibacter* strains shared 96.7–100% of amino acid identity with each other, placing all the isolates as members of the same genus. The highest AAI values, when *Mergibacter* was compared with other representative members of the *Pasteurellaceae* family, were observed with species of the genus *Gallibacterium* (ranging 67–70%). Thus, the AAI values were below the genus threshold, further supporting the genus identity of *Mergibacter*. Along with AAI, the percentage of conserved protein (POCP)-based taxonomic test has been demonstrated to be a robust tool for establishing the genus boundary, set to below 50% for most prokaryotic groups (Qin et al., 2014; Barco et al., 2020). POCP values between *Mergibacter* and its closest genetic species (based on our core genome phylogeny) were above the 50% threshold (i.e., 68.34% with nearest neighbor *G. genomosp. 3*). However, the POCP values observed between the other *Pasteurellaceae* members included in the analysis were never lower than 63%, as observed in the POCP study between *Chelonobacter* and *Gallibacterium*, suggesting that the 50% threshold may not be reliably applied to the *Pasteurellaceae* family. An additional investigation involving different genera belonging to the *Pasteurellaceae* family may be helpful to assess a reliable POCP-based genus boundary value for this specific group of bacterial pathogens. A new taxonomic approach to defining a genus boundary within the *Pasteurellaceae* family based on an AAI method using only 31 randomly selected CDS was described (Christensen and Bisgaard, 2018). The authors concluded that *Pasteurellaceae* isolates sharing 88% amino acid pairwise similarity belonged to the same genus, establishing a 88% genus boundary within the *Pasteurellaceae* family (Christensen and Bisgaard, 2018). In this study, the highest similarity percentage observed between *Mergibacter* and the other *Pasteurellaceae* strains was 69.59%, well below the 88% genus boundary value (Christensen and Bisgaard, 2018). Similar to (Christensen and Bisgaard, 2018), an exception was found between *Chelonobacter* and *P. testudinis*, which shared values above the 88% threshold. However, they are currently classified into two different genera within the *Pasteurellaceae* family. These findings indicate that a reclassification for some *Pasteurellaceae* members may be warranted, as similarly stated by (Christensen and Bisgaard, 2018).

Sequence comparison based on 16S rRNA and housekeeping genes has been deemed suitable for phylogenetic inference due to these genes’ evolutionary conservation and low selection pressure (Christensen et al., 2004). Indeed, within the *Pasteurellaceae* family, published minimal standards for describing a new genus require a monophyletic grouping based on 16S rRNA gene sequences and a 16S rRNA gene sequence divergence greater than ~5% from other recognized genera (Christensen et al., 2007). Based on the 16S rRNA gene sequence analysis, the five *M. septicus* strains showed sequence similarities among each other greater than 99.5%, while the highest nucleotide identities that were found between *Mergibacer* and other *Pasteurellaceeae* species never went above 92.2%. Additionally, the monophyly of genera within the *Pasteurellaceae* has been confirmed by sequence comparison of *infB* and *rpoB* genes with members of the same genus showing similarity values of 85–88% or higher at the nucleotide sequence level (Christensen et al., 2007). Here, we further investigated the sequence similarity of the *Merigbacer* strains with an MLS that comprised the core portion of housekeeping genes *atpD, infB*, *recN, and rpoB*. Once again, *M. septicus* showed sequence similarities among each other above 99%, while the highest nucleotide identities found between *Mergibacer* strains and other *Pasteurellaceeae* species never reached 85%. This further evidence the correct classification of *Mergibacer* as a new genus.

The tree’s topology resulting from the MLS-based phylogeny was very similar to the topology of the tree estimated using *Pasteurellaceae* core genomes, placing genera *Mergibacter Gallibacterium Pasteurella*, *Chelonobacter*, *Testudinibacter*, and *Spirabiliibacterium* as well-differentiated terminal groups of a more extensive common clade. Surprisingly, this was not observed in the 16S tree where *Mergibacter* appeared at a considerable genetic distance from the *Gallibacterium* species. Additionally, the unreliability of the 16S-based taxonomy for this family is also confirmed by the inconsistent clustering of members belonging to the *Pasteurella* genus observed in the 16S phylogenetic tree and the lack of bootstrap support. These two factors highly improved in the MLS-based phylogenetic analysis; nevertheless, two genera appeared to be misclassified. This indicates that, although not perfect, the four housekeeping genes employed in this study better represent the *Pasteurellacea*e whole genomes, and their phylogenetic relatedness is more accurately described using our MLS approach than when exclusively using the 16S rRNA gene. Overall, our study highlights that a reclassification for some *Pasteurellaceae* members is needed using new and more robust taxonomical approaches.

Pathogenicity in *Pasteurellaceae* species and subspecies is associated with various virulence factors that critically contribute to colonization and invasion of the host (Ewers et al., 2006). Most of the *M. septicus* strains characterized in this study originated from mortality events of wild birds with evidence of septicemia (Knowles et al., 2019; Niedringhaus et al., 2021). Our *in silico* genetic characterization revealed that *M. septicus* carries up to twelve major virulence factors associated with disease in other avian pathogens of the family Pasteurellaceae, including genes that encode a putative capsule (capsule), outer membrane proteins (*ompA, ompH*), a cytolethal distending toxin (*cdt*), iron metabolic proteins (*exbD, fur*), a superoxide dismutases (*sodA*), and proteins involved in lipooligosaccharide (LOS) synthesis (*galU, galE, lpxA, lpxC, kdsA*). Indeed, the presence of a capsule gene homolog to the one identified in *M. septicus* has been correlated to increased virulence in *Pasteurella multocida* because encapsulated organisms are more resistant to phagocytosis and to the bactericidal activity of complement (Boyce et al., 2000). Similarly, the putative virulence genes found in *Margibacter* are homologs to outer membrane protein encoding genes *ompA* and *ompH*. It has been described that *ompA* and *ompH* mediate *P. multocida*-host cells interaction *via* heparin and/or fibronectin binding and therefore act as an important invasive factors which might determine the outcome of initial infection (Katoch et al., 2014). Furthermore, the iron metabolism-related genes homologs to the ones found in *Mergibacter* are associated with the iron uptake from transferrin proteins (Magarinos et al., 1994), while the superoxide dismutase enzymes neutralize toxic levels of reactive oxygen species generated by the host cell, thus contributing to the virulence (Cox et al., 2003). Contrary to previous studies, which have widely demonstrated that adhesins are frequently among pathogenic isolates of *Pasteurella multocida*, no adhesin genes were identified in *Mergibacter* strains. Instead, several genes involved in lipooligosaccharide production were detected. Intriguingly, all the strains of this study carried a *cdt* gene encoding the cytolethal distending toxin proven to cause cell cycle arrest and death in eukaryotic cells (Elwell and Dreyfus, 2000; Lara-Tejero and Galan, 2000). Except for *Avibacterium paragallinarum* (Chen et al., 2014), *M. septicus* represents the only *Pasteurellaceae* member known to carry this virulence factor, indicating a role in disease outbreak. For most of the shared virulence genes, the phylogenetic relationships of *Mergibacter* with *Pasteurellaceae* members were investigated, and all the resultant phylogenetic trees performed showed its taxonomic position in a distinct branch within the *Pasteurellaceae* family (Figure 6), supporting the reclassification of *Mergibacter* as a novel genus of this family.

Antibiotic therapy is an effective tool in treating infections caused by *Pasteurellaceae* in birds; however, an increased occurrence of multidrug-resistant strains has been widely described in the past decade (Furian et al., 2016). In this study, the *Mergibacter* type strain *M. septicus* A25201 demonstrated low MICs for all the antibiotics tested, and no ARGs or ARGs-carrying plasmids were observed in the *Mergibacter* genome. Additional antimicrobial susceptibility data and clinical outcomes from treatments of seabirds would be helpful to implement specific antimicrobial susceptibility recommendations, as no specific clinical breakpoints are currently in place.

The major limitation of this study is the small number of isolates investigated. Evaluation of a more significant number of isolates may be helpful to examine whether diversity in virulence factors is associated with disease prevalence and severity and ascertain the taxonomic position of this bacterium. Additionally, for the type strain A25201 (DSM 112696; CP054053), we used a combination of short and long-read sequencing as an assembly approach, which is considered the method of choice for assembling plasmids (Margos et al., 2017). However, the step of lysing bacterial cells represents a critical phase in DNA extraction, and the quality of plasmid DNA recovered is primarily determined in this step (Zhu et al., 2006). Because we did not use a specific plasmid extraction kit, the loss and/or fragmentation of plasmids carrying antimicrobial resistance genes and/or virulence factors during the DNA extraction process cannot be ruled out.

## Conclusion

We describe the first whole-genome sequence characterization for Bisgaard taxon 40 strains implicated in avian disease and mortality events. Our results provide a better understanding of the phylogenetic relationships among the *Pasteurellaceae*. They indicate that Bisgaard taxon 40, although closely related to the *Gallibacterium* genus, warrants classification as a new genus within the *Pasteurellaceae* family, and it was renamed as *Mergibacter* with the type species *M. septicus* A25201 (DSM 112696; CP054053). A25201 demonstrated considerably low MICs for all the drugs tested, including penicillin, amoxicillin, and tetracycline, which may represent potential therapeutic suggestions. Furthermore, this study provides insights into the pathogenic potential of *M. septicus*, highlighting that virulence factors involved in the adhesion and colonization of the host, as well as the *cdt* toxin, may have played a significant role in the disease development in several seabird species and contributed to mass mortality events. This study provides a genomic characterization and comparative analysis of this bacterium, highlighting the importance of advanced biotechnologies, such as whole-genome sequencing, as an essential tool to characterize and unravel the complex phylogeny of the *Pasteurellaceae* members. We also demonstrated the usefulness of MLS-based phylogenetic analysis as a reliable approach to distinguish *Mergibacter* from other genera of the *Pasteurellaceae* family. Although 16S RNA-based taxonomy is sufficient to separate *Mergibacter* from other *Pasteurellaceae* genera, this study provides insights into the limitations of this method to accurately classify the *Pasteurellaceae*. Our novel MLS method appears to be a cost-effective alternative to 16S rRNA, especially in case of limited access to whole-genome sequencing resources. Given *Mergibacter* ability to infect avian species and its association with large-scale mortality events, we posit that the public health community should maintain vigilance against and awareness of the unusual clinical, pathological, and microbiological presentation of this microorganism.

## Protologue

### Description of Mergibacter gen. nov.

*Mergibacter* (Mer.gi.bac’ter. L. masc. n. mergus a seabird; N.L. masc. n. bacter a rod; N.L. masc. n. Mergibacter a rod of a seabird). *Mergibacter* is a new genus within the *Pasteurellaceae* family. The cells are Gram-negative staining rods, and no flagella or pili have been observed. *Mergibacter* was isolated on 5% sheep blood agar after 48h of aerobic incubation at 42°C, while no growth was observed on MacConkey. The colonies were β-hemolytic, shiny, circular, and whitish-cream. Positive reactions were observed for oxidase, catalase; negative tests were observed for urease and indole. Acid was formed from maltose and dextrin but not from D-mannitol, L-fucose, sucrose, and trehalose. A comparison of phenotypic characters separating the genus *Mergibacter* from the other *Pasteurellaceae* is given in Table 2. *Mergibacter septicus* is the type species of the genus.

### Description of *Mergibacter septicum* sp.nov.

*Mergibacter septicus* (sep’ti.cus. L. masc. Adj. septicus septic). In addition to characteristics included in the genus description, acid is formed from D-sorbitol and D-galactose. Most of the strains, including the type strain, are able to produce acid from D-mannose. Isolates have mainly been obtained from internal organs, such as the heart, lung, spleen, and brain.

The type strain is *Mergibacter septicus* A25201^T^ (DSM 112696), isolated from the heart and liver of one common tern (*Sterna hirundo*) from a mortality event in Florida, United States. In addition to the characteristics described above, using GEN III Microbial ID acid was formed from D-maltose, D-cellobiose, N-acetyl-D-Glucosamine, N-acetyl-β-D-Mannosamine, N-Acetyl-Neuraminic acid, D-Glucose, D-Mannose, D-Sorbitol, D-Glucose6-PO4, D-Fructose6-PO4, and acetoacetic acid. The genome of the type strain is 1,887,770bp long with a G+C content of 36.4%. The genome sequence is deposited in GenBank under the accession number CP054053.1.

### Deposit in Culture Collections

The type strain A25201^T^ (*Mergibacter septicus* sp.; nov.) has been deposited in the DSMZ (German collection of microorganisms and cell cultures) under the number DSM 112696. In addition deposit at ATCC is in progress, AcqID-003043.

## Data Availability Statement

The datasets presented in this study can be found in online repositories. The names of the repository/repositories and accession number(s) can be found in the article/Supplementary Material.

## Author Contributions

EL: formal analysis, investigation, methodology, bioinformatic analysis, writing-original draft, and writing-review and editing. SÁ-N: formal analysis, investigation, bioinformatic analysis, and writing-review and editing. GM: formal analysis, investigation, methodology, and writing-review and editing. RB: bioinformatic analysis and writing-review and editing. NN: writing-review and editing. KN: writing-review and editing. JLa: formal analysis, methodology, supervision, and writing-review and editing. JLo: methodology and writing-review and editing. GK: investigation and writing-review and editing. SL: formal analysis and writing-review and editing. GP: funding acquisition, supervision and writing-review and editing. SS: conceptualization, methodology, funding acquisition, investigation, resources, supervision, and writing-review and editing. All authors contributed to the article and approved the submitted version.

## Funding

This work was funded by the Athens Veterinary Diagnostic Laboratory, Department of Infectious Diseases, University of Georgia, Georgia, United States of America and a grant from the FDA to SS 5U18FD006157. EL was supported by the College of Veterinary Medicine at the University of Georgia and Boehringer Ingelheim. The use of trade, firm, or product names is for descriptive purposes only and does not imply endorsement by the U.S. Government. The work was funded in part by the Defense Threat Reduction Agency.

## Conflict of Interest

The authors declare that the research was conducted in the absence of any commercial or financial relationships that could be construed as a potential conflict of interest.

## Publisher’s Note

All claims expressed in this article are solely those of the authors and do not necessarily represent those of their affiliated organizations, or those of the publisher, the editors and the reviewers. Any product that may be evaluated in this article, or claim that may be made by its manufacturer, is not guaranteed or endorsed by the publisher.

## Footnotes

^1^ https://github.com/rrwick/Porechop

^2^ http://tree.bio.ed.ac.uk/software/figtree

^3^ https://www.ncbi.nlm.nih.gov/

^4^ https://card.mcmaster.ca/analyze/rgi

^5^ https://cge.cbs.dtu.dk/services/ResFinder/

^6^ https://www.ncbi.nlm.nih.gov/pathogens/antimicrobial-resistance/AMRFinder/

^7^ https://cge.cbs.dtu.dk/services/PlasmidFinder

^8^ https://castillo.dicom.unican.es/copla/

## Supplementary Material

**Supplementary Figure S1 |** *Mergibacter septicus* A25201^T^ (=DSM 112696) grown on blood agar. Growth of pure colonies after 48h of incubation at 42°C. Bacterial colonies appeared as β-hemolytic, shiny, circular and whitish-cream.

**Supplementary Figure S2 |** Virulence factors detected in *Mergibacter septicus* strains. The heatmap is based on the similarity of each virulence gene for five *Mergibacter septicus* strains. It was generated using GraphPad Prism Version 8.4.3. The color key on the right indicates the percentage of gene similarity.
