# Supplementary material for: Comparative Genomics Analyses Support the Reclassification of Bisgaard Taxon 40 as Mergibacter gen. nov., With Mergibacter septicus sp. nov. as Type Species: Novel Insights Into the Phylogeny and Virulence Factors of a Pasteurellaceae Family Member Associated With Mortality Events in Seabirds
Source: Front Microbiol. 2021 Nov 22;12:667356. doi: 10.3389/fmicb.2021.667356 (PMC8645869; doi:10.3389/fmicb.2021.667356)
Supplement: Supplementary file 1 [file Table_1.docx]

**Supplementary Tables.**

**Table S1.** Five *Mergibacter septicus* strains and 58 representative *Pasteurellaceae* genomes from the NCBI database were used in the housekeeping genes-based and 16S RNA gene-based analyses. * Genome sequences included in the whole genome-based phylogenetic tree.

| Scientific name | Strain (n, =5) | GenBank accession no. | Genus type strain  (n, =1) | Species reference genome  (n, =1) |
| --- | --- | --- | --- | --- |
| **Mergibacter septicus* | A25201 DSM 112696 | CP054053 | yes | yes |
| **Mergibacter septicus* | 27643 | CP022010 | no | no |
| **Mergibacter septicus* | 27576 | CP022011 | no | no |
| **Mergibacter septicus* | 16309 | CP022012 | no | no |
| *Mergibacter septicus* | 27517 | CP022013 | no | no |
| Scientific name | **Strain (n, = 58)** | **GenBank accession no.** | **Genus type strain**  **(n, = 37)** | **Species reference genome**  **(n, = 46)** |
| *Actinobacillus lignieresi* | NCTC 4189 | UFRM01 | yes | yes |
| *Actinobacillus suis* | ATCC 33415 | CP009159 | no | yes |
| **Actinobacillus suis* | NCTC 12966 | LT906456 | no | no |
| **Aggregatibacter actinomycetemcomitans* | 624 | CP012959 | yes | no |
| *Aggregatibacter actinomycetemcomitans* | RhAA1 | JPZI01 | yes | yes |
| **Avibacterium avium* | NCTC 11297 | UGSP01 | no | yes |
| **Avibacterium endocarditis* | 20186H4H1 | PQVI01 | no | yes |
| *Avibacterium gallinarum* | DSM 17481 | SNXJ01 | yes | yes |
| **Avibacterium gallinarum* | NCTC 11188 | UGSQ01 | yes | no |
| *Avibacterium paragallinarum* | ESV-135 | CP050316 | no | yes |
| **Avibacterium paragallinarum* | JF4211 | CBMK01 | no | no |
| **Avibacterium volantium* | NCTC 3438 | LR134167 | no | yes |
| **Basfia succiniciproducens* | JF4016 | CP015031 | yes | yes |
| *Biberstenia trehalosi* | USDA-ARS-USMARC-188 | CP006954 | yes | yes |
| **Biberstenia trehalosi* | Y31 | JACI01 | yes | no |
| **Bisgaardia hudsonensis* | M327/99/2 | CP016605 | yes | yes |
| *Canicola haemoglobinophilus* | NCTC 1659 | UGHF01 | yes | yes |
| **Caviibacterium pharyngocola* | 7.3 | PHGZ01 | yes | yes |
| **Chelonobacter oris* | 1662T | JSUM01 | yes | yes |
| **Conservatibacter flavescens* | 7.4 | PHHA01 | yes | yes |
| *Cricetibacter osteomyelitidis* | DSM 28404 | SLYB01 | yes | yes |
| **Frederiksenia canicola* | HPA 21 | CP015029 | yes | yes |
| **Gallibacterium anatis* | NCTC 11413 | UGGZ01 | yes | yes |
| **Gallibacterium genomosp. 1* | CCM5974 | JPXX01 | no | yes |
| **Gallibacterium genomosp. 2* | CCM5976 | JPXY01 | no | yes |
| **Gallibacterium genomosp. 3* | 59/63/89 | JTJR01 | no | yes |
| **Gallibacterium salpingitis* | F150 | JTJL01 | no | yes |
| **Glaesserella parasuis* | SH0165 | NC_011852 | yes | no |
| *Glaesserella parasuis* | YHP1818 | CP071487 | yes | yes |
| *Haemophylus influenzae* | 65290_NP_Hi3 | QWLX01 | no | yes |
| **Haemophylus influenzae* | NCTC 8143 | LN831035 | yes | no |
| **Histophilus somni* | 2336 | CP000947 | yes | no |
| *Histophilus somni* | USMARC-63368 | CP018804 | yes | yes |
| **Lonepinella koalarum* | DSM 10053 | SMGJ01 | yes | yes |
| **Mannheimia haemolytica* | M42548 | CP005383 | yes | no |
| *Mannhemia haemolytica* | USMARC_2286 | CP006619 | yes | yes |
| **Mesocricetibacter intestinalis* | DSM 28403 | SNYQ01 | yes | yes |
| **Muribacter muris* | 7 | JADGLQ01 | yes | yes |
| **Necropsobacter massiliensis* | FF6 | CDON01 | no | yes |
| *Necropsobacter rosorum* |  | CCMQ01 | yes | yes |
| **Nicoletella semolina* | DSM 16380 | SLXJ01 | yes | yes |
| **Otariodibacter oris* | Baika1 | CP016604 | yes | yes |
| **Pasteurella langaaensis* | DSM 22999 | QENU01 | no | yes |
| *Pasteurella multocida* | 20N | CP028926 | yes | yes |
| *P. multocida subsp. multocida* | ATCC 43137 | CP008918 | no | no |
| **P. multocida subsp. gallicida* | NCTC 10204 | LR134298 | no | yes |
| **P. multocida subsp. septica* | CIRMBP-0873 | CP020347 | no | no |
| *P. multocida subsp. septica* | NCTC 10322 | UGSW01 | no | yes |
| **Pasteurella testudinis* | DSM 23072 | FWWV01 | no | no |
| **Pasteurella testudinis* | NCTC 12150 | UGSY01 | no | yes |
| **Phocoenobacter uteri* | NCTC 12872 | UGTA01 | yes | yes |
| *Rodentibacter heylii* | G1 | CP040863 | no | yes |
| *Rodentibacter pneumotropicus* | ATCC 35149 | BBIX01 | yes | yes |
| **Spirabiliibacterium falconis* |  | _JABMIJO1 | yes | yes |
| **Testudinibacter aquarius* | DSM 28140 | SMCP01 | yes | yes |
| *Ursidibacter maritimus* | Pb43106 | LEKN01 | yes | yes |
| **Vespertiliibacter pulmonis* | CCUG 64585 | CP016615 | yes | yes |
| **Volucribacter psittacicida* | DSM 15534 | SMFT01 | yes | yes |

**Table S2.** Additional 16S rRNA gene sequences downloaded from NCBI and included in the 16S rRNA-based phylogenetic analysis.

| Scientific name | Strain | GenBank accession no. |
| --- | --- | --- |
| *Actinobacillus anseriformium* | biovar 1 F66 | NR_115157 |
| *Actinobacillus anseriformium* | biovar 2 F97 | AY172728 |
| *Actinobacillus anseriformium* | CCUG 28015 | AF224284 |
| *Actinobacillus arthritidis* | CCUG 24862 | NR_115218 |
| *Avibacterium paragallinarum* | NCTC 11296 | NR_042932 |
| *Bisgaard Taxon 34* | 69 | AY172731 |
| *Gallibacterium anatis* | DSM 16844 | NR_036870 |
| *Gallibacterium melopsittaci* | F450 | NR_116281 |
| *Gallibacterium salpingitidis* | 18469/18 | EU339205 |
| *Gallibacterium trehalosifermentans* | 52/S3/90 | NR_044470 |
| *Mergibacter septicus* | B301529/00/1 | AY172732 |
| *Pasteurella langaaensis* | ATCC 43328 | M75053 |
| *P. multocida subsp. gallicida* | HIM830-7T | AF326323 |
| *Spirabiliibacter mucosae* | P40 | AY172725 |
| *Spirabiliibacter mucosae* | CCUG 16499 | AY362901 |
| *Spirabiliibacter mucosae* | CCUG 28027 | L06087 |
| *Spirabiliibacter pneumonia* | HPA106 | AY172729 |
| *Volucribacter amazonae* | 146/S8/89 | NR_042796 |
| *Volucribacter psittacicida* | Gerl 236/81 | NR_025724 |

**Table S3.** Percentage of conserved protein (POCP) obtained using the method published by Quin and collaborators (Qin et al., 2014). POCP of 50% are considered the genus boundary for some prokaryotic groups.

|  | *Mergibacter septicus* | *Gallibacterium genomosp. 3* | *Chelonobacter oris* | *Pasteurella testudinis* |
| --- | --- | --- | --- | --- |
| *Mergibacter septicus* |  | 68.34221884 | 63.28182487 | 60.818449 |
| *Gallibacterium genomosp. 3* | **68.3422188** |  | 68.7337943 | 63.8922396 |
| *Chelonobacter* | **63.2818249** | 68.7337943 |  | 79.3231612 |
| *Pasteurella testudinis* | **60.818449** | 63.89223965 | 79.32316119 |  |

**Table S5.** Similarity values based on the predicted conserved protein sequences from the 31 genes (Christensen and Bisgaard, 2018). 88% protein pairwise similarity was established as genus boundary.

|  | *Chelonobacter* | *Pasteurella testudinis* | *Mergibacter septicus* | *Gallibacterium anatis* | *Gallibacterium genomosp. 2* | *Gallibacterium genomosp. 1* | *Gallibacterium genomosp. 3* | *Gallibacterium salpingitis* |
| --- | --- | --- | --- | --- | --- | --- | --- | --- |
| *Chelonobacter* |  | 91.87 | **67.82** | 75.46 | 75.41 | 75.29 | 75.82 | 75.86 |
| *Pasteurella testudinis* | 91.87 |  | **68.2** | 75.44 | 75.29 | 75.29 | 75.76 | 75.87 |
| *Mergibacter septicus* | 67.82 | 68.2 |  | 69.46 | 69.33 | 69.37 | 69.59 | 69.77 |
| *Gallibacterium anatis* | 75.46 | 75.44 | **69.46** |  | 97.43 | 97.21 | 89.16 | 89.18 |
| *Gallibacterium genomosp. 2* | 75.41 | 75.29 | **69.33** | 97.43 |  | 98.34 | 88.77 | 88.69 |
| *Gallibacterium genomosp. 1* | 75.29 | 75.29 | **69.37** | 97.21 | 98.34 |  | 88.89 | 88.7 |
| *Gallibacterium genomosp. 3* | 75.82 | 75.76 | **69.59** | 89.16 | 88.77 | 88.89 |  | 93.73 |
| *Gallibacterium salpingitis* | 75.86 | 75.87 | **69.37** | 89.18 | 88.69 | 88.7 | 93.73 |  |
