# Supplementary figures and images for: Comparative Genomics Analyses Support the Reclassification of Bisgaard Taxon 40 as Mergibacter gen. nov., With Mergibacter septicus sp. nov. as Type Species: Novel Insights Into the Phylogeny and Virulence Factors of a Pasteurellaceae Family Member Associated With Mortality Events in Seabirds
Source: Front Microbiol. 2021 Nov 22;12:667356. doi: 10.3389/fmicb.2021.667356 (PMC8645869; doi:10.3389/fmicb.2021.667356)

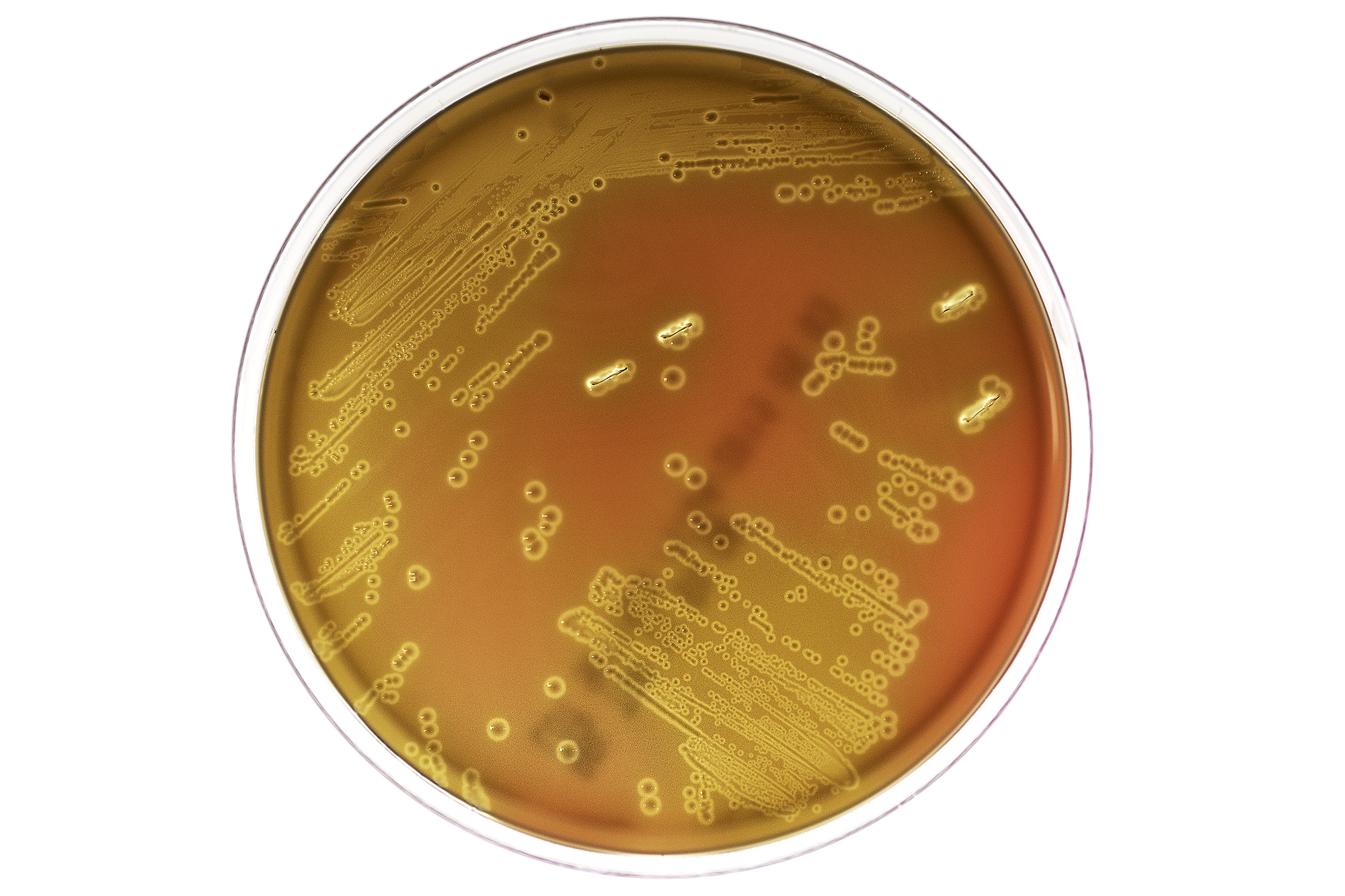

Supplement: Supplementary file 2 [file Image_1.TIF]

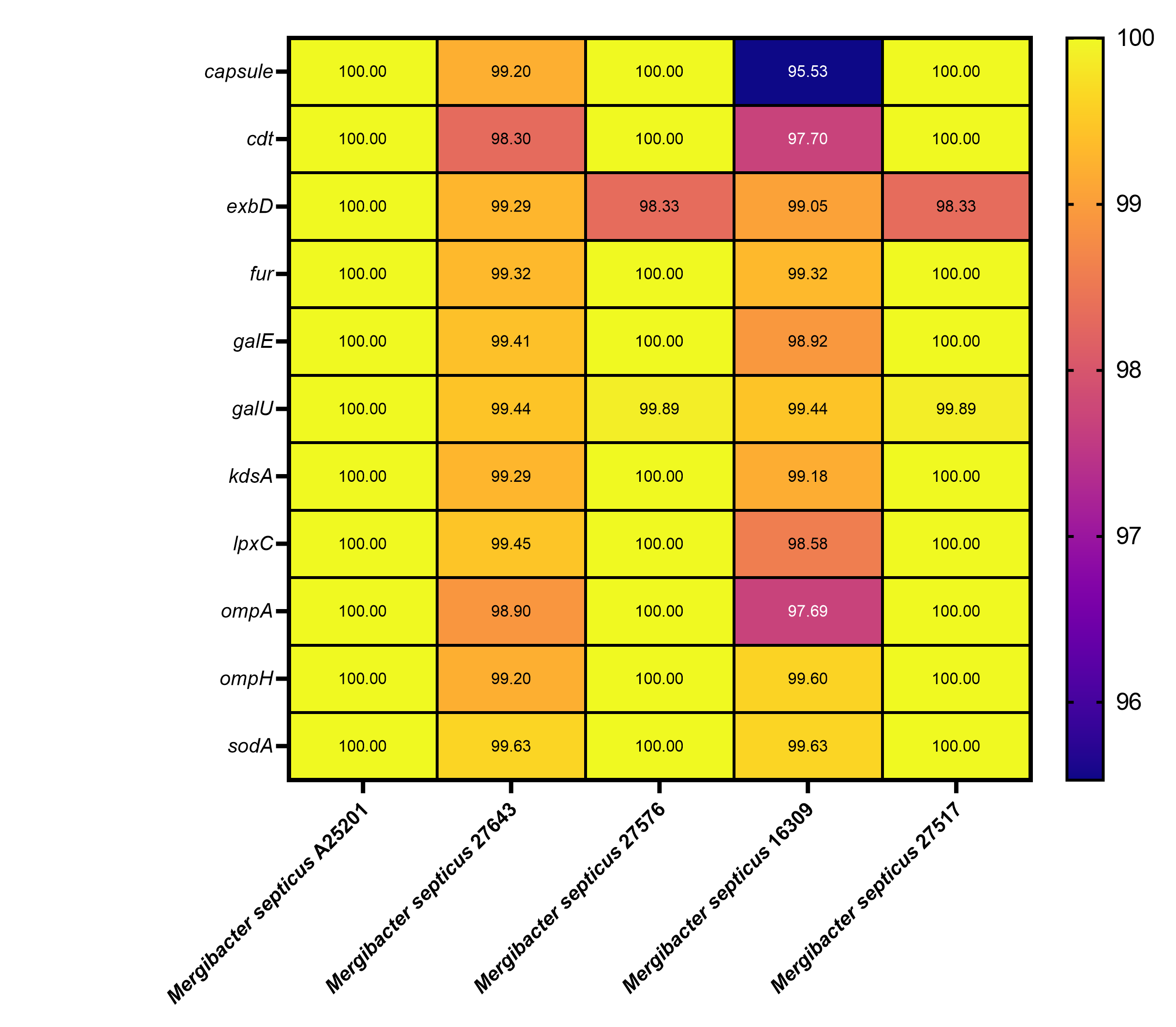

Supplement: Supplementary file 3 [file Image_2.TIF]
